# Supplementary figures and images for: Potential for online crowdsourced biological recording data to complement surveillance for arthropod vectors
Source: PLoS One. 2021 Apr 30;16(4):e0250382. doi: 10.1371/journal.pone.0250382 (PMC8087023; doi:10.1371/journal.pone.0250382)

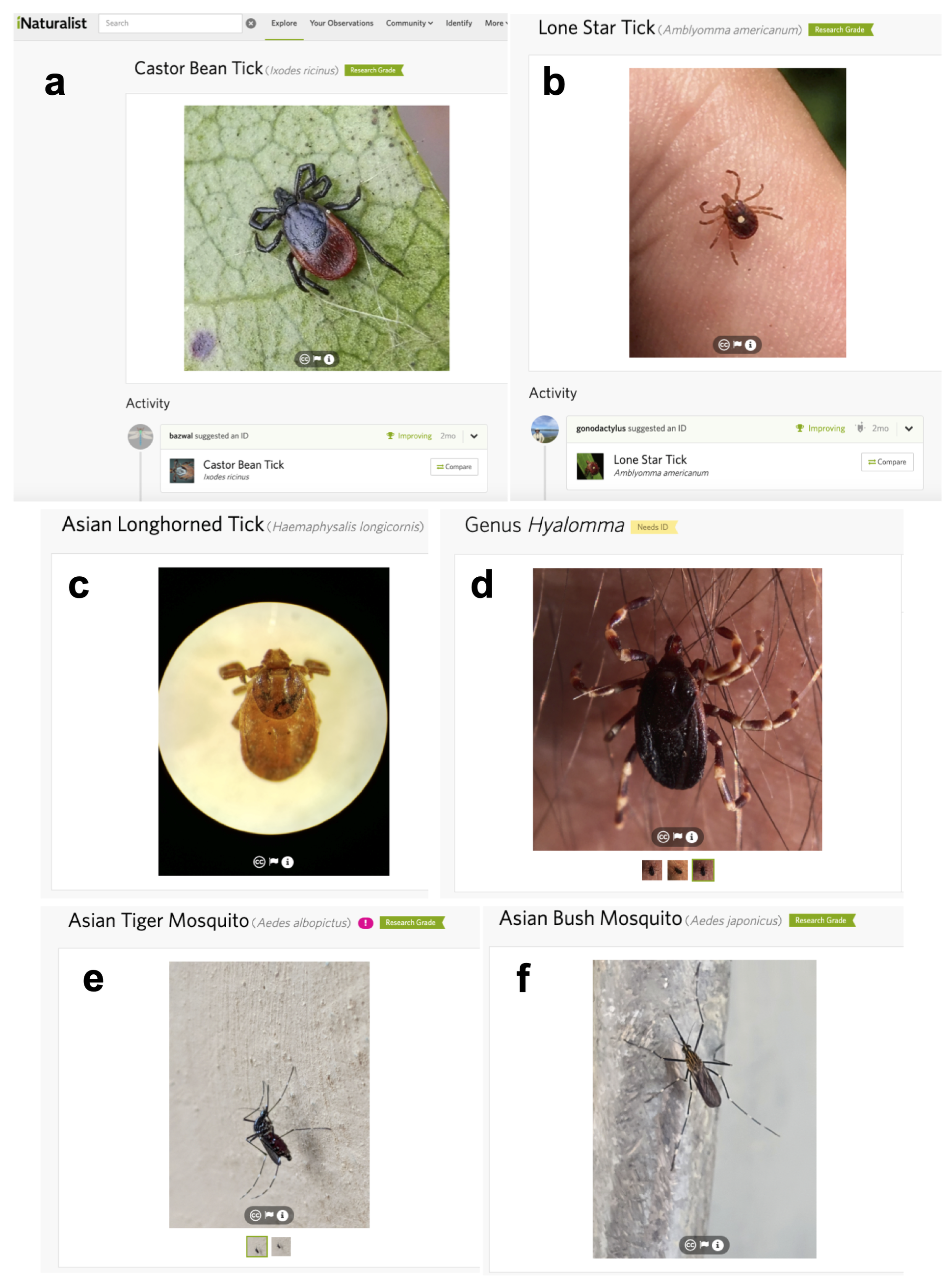

Supplement: S1 Fig — (a) case study 1, Ixodes ricinus recorded in the UK; (b) case study 2, Amblyomma americanum recorded in Minnesota, USA; (c) case study 4, record of Haemaphysalis longicornis from New York state, USA; (d) case study 3, Hyalomma spp. recorded in Spain; (e) case study 5, Aedes albopictus observed in Italy; (f) case study 5, Aedes japonicus observed in Austria. These images were taken by iNaturalist users and are made available under Creative Commons license (CC BY 4.0). (TIF) [file pone.0250382.s001.tif]
